# Perceptions and practices of mosquito-borne diseases in Alabama – is concern where it should be?

**DOI:** 10.1186/s12889-019-7308-x

**Published:** 2019-07-23

**Authors:** Wayde Morse, Katie Izenour, Benjamin McKenzie, Sarah Lessard, Sarah Zohdy

**Affiliations:** 1 Auburn University School of Forestry and Wildlife Sciences, 602 Duncan Dr, Auburn, AL 36849 USA; 2 Auburn University College of Veterinary Medicine, 166 Greene Hall, Pathobiology Rm 161, Auburn, AL 36849 USA

**Keywords:** KAP survey, Zika, Chikungunya, West Nile virus, Malaria, Vector control, Mosquito ecology

## Abstract

**Background:**

The Gulf Coast of the United States is home to mosquito vectors that may spread disease causing pathogens, and environmental conditions that are ideal for the sustained transmission of mosquito-borne pathogens. Understanding public perceptions of mosquito-borne diseases and mosquito prevention strategies is critical for the development of effective vector control strategies and public health interventions. Here, we present a survey conducted in Mobile, Alabama along the Gulf Coast to better understand public perceptions of mosquito-borne diseases, mosquito control activities, and potential risk factors.

**Methods:**

Using Knowledge, Attitude, and Practice (KAPs) assessments, we surveyed populations living in 12 zip codes in Mobile, Alabama using a 7-point Likert scale and frequency assessments. Survey participants were asked about vector control efforts, knowledge of mosquito-borne diseases, and understanding of mosquito ecology and breeding habitats.

**Results:**

One hundred twenty-six surveys were completed in Mobile, Alabama, revealing that 73% of participants reported being bitten by a mosquito in the last 30 days and mosquitoes were frequently seen in their homes. Ninety-four percent of respondents had heard of Zika Virus at the time of the survey, and respondents reported being least familiar with dengue virus and chikungunya virus.

**Conclusions:**

Chikungunya virus, dengue virus, malaria, West Nile virus, and Zika virus have been documented in the Gulf Coast of the United States. The mosquitoes which vector all of these diseases are presently in the Gulf Coast meaning all five diseases pose a potential risk to human health. The results of this survey emphasize knowledge gaps that public health officials can address to empower the population to reduce their risk of these mosquito-borne diseases. Each species of mosquito has specific preferences for breeding and feeding and there is no one size fits all prevention approach, educating people on the need for a variety of approaches in order to address all species will further empower them to control mosquitoes where they live and further reduce their risk of disease.

**Electronic supplementary material:**

The online version of this article (10.1186/s12889-019-7308-x) contains supplementary material, which is available to authorized users.

## Background

The sub-tropical climate of the US Gulf Coast, as well as an abundance of habitat for *Aedes, Culex* and *Anopheles* mosquitoes and the proximity of trade and travel hubs, put the Gulf Coast at risk for the establishment or re-establishment of mosquito-borne pathogens in the US [1]. Until the implementation of vigorous vector control efforts in the mid-twentieth century, the Gulf Coast experienced regular outbreaks of malaria and yellow fever [2, 3]. In more recent years, the Gulf Coast has been under threat of endemic transmission of mosquito-borne RNA viruses such as West Nile virus (WNV), dengue fever virus (DENV), chikungunya virus (CHIKV) and Zika virus (ZIKV) [4–7]. Vector control efforts have been stymied by the growing prevalence of insecticide resistance among many mosquito taxa as well as funding cuts due to a public perception that mosquito-borne pathogens no longer present much threat in the US [8, 9]. The potential for emergence and re-emergence of mosquito-borne diseases in this geographic region points to a growing need to develop strategies to combat the transmission of mosquito-borne pathogens.

A few mosquito-borne pathogens that pose potential threats in the region due to the distribution of their mosquito vectors include CHIKV, DENV [10], malaria (MAL) [11], WNV [12, 13] and ZIKV. All of these pathogens have been detected in Gulf Coast states in recent years, though many of these cases have been considered to be imported cases, not acquired locally (Table 1). WNV, which is transmitted by *Culex* mosquitoes, is the most prevalent mosquito-borne disease in the US, with an average of 2,000 and up to 9,800 cases per year (between 1999 and 2017) [16]. Symptoms range from mild febrile illness, to severe encephalopathy to death [17]. Meanwhile, ZIKV, DENV and CHIKV, all transmitted by *Aedes* mosquitoes, have recently emerged and re-emerged in Gulf States, mostly in travel-related cases, but achieving autochthonous transmission in Florida [5, 6, 18]. Historically, MAL was prevalent in the southeastern US, with parasites transmitted by *Anopheles* mosquitoes. Malaria symptoms typically include cyclical fevers, anemia, and fatigue and are often fatal in pregnant women and children under the age of 5 [19]. Following massive mosquito control efforts in the US, including the use of DDT as pesticide, MAL was eliminated from the region [2]. While MAL transmission is not endemic to the southeastern US, there are still a handful of reported human cases annually, typically attributed to travel. A well-known mosquito vector of MAL (*An. quadrumaculatus*) remains around the US Gulf Coast and is a very common nocturnal mosquito. It is important to note that these pathogens are transmitted by different genera of mosquitoes, each with different ecologies and thus requiring different strategies for vector control (Table 2).

**Table 1:** Distribution and risk of mosquito-borne diseases that have been reported along the Gulf Coast and Alabama [5, 14, 15]

Disease	Reported in Alabama	Reported along Gulf Coast	*Total No. Cases in Alabama 2013–2018
chikungunya virus	Imported cases	Locally acquired	22
dengue virus	Imported cases	Locally acquired	17
malaria	Imported cases	Imported cases	53
West Nile virus	Locally acquired	Locally acquired	128
zika virus	Imported cases	Locally acquired and imported	49

**Table 2:** Important characteristics of mosquito species found in Alabama

Genus	Species	Vectors of Disease	Active period	Breeding	Resilience
*Aedes*	*aegypti albopictus*	Zika virus, dengue fever virus, chikungunya virus	Daytime	Tires, streams, puddles	Eggs can survive without water 6 months
*Anopheles*	*quadrimaculatis*	Malaria	Nighttime	Agricultural fields	Eggs require water for survival
*Culex*	*pipiens tarsalis quinquefasciatus*	West Nile virus		Wastewater, sewage, bird baths, flower pots	Eggs require water for survival

While many public health strategies exist to combat the spread of mosquito-borne pathogens, including insecticide use, gene drives and reduction of breeding habitat, none of these methods is entirely effective on its own and all of these methods benefit from the aid of public education [20–22]. A populace educated in the transmission cycles of mosquito-borne pathogens, as well as common steps taken to interrupt this cycle, can be invaluable to public health efforts [20, 21]. Thus, an informed private citizenry could be an important tool for vector control strategies in the region.

To gauge residents’ current level of understanding surrounding mosquito-borne pathogens present in the Gulf Region, we conducted a pilot survey among residents of the Mobile Bay area in the Gulf, where *Ae. aegypti* mosquitoes, which are responsible for mosquito borne diseases like CHIKV, ZIKV, and DENV have recently been detected following a 26-year absence [1]. Through this pilot survey, we asked residents of Alabama’s Mobile Bay area about their practices to reduce the number of mosquitoes, level of knowledge, degree of concern and perceptions of seriousness about five mosquito-borne diseases, preferred modality for receiving information and the perceived impact their mosquito prevention measures have on their homes and neighborhood. This information is the core of planning and targeting public health and community prevention and outreach programs. We hypothesize that residents of the Mobile area will report being bitten frequently by mosquitoes. We also hypothesize that Mobile area citizens will report high levels of knowledge and concern about ZIKV and less knowledge and concern about the other diseases that are of equal or greater importance in the area.

## Methods

### Survey and sample selection

A fifty-question paper survey developed for this project was mailed to a random sample of 1,000 residents in the urban center of Mobile Bay along Alabama’s Gulf Coast (Additional file 1). Surveys were sent out in July and August 2016 along with two follow-up reminders; a post card and a letter spaced two weeks apart. Best practices in survey design and development were followed [23]. The survey asked questions about the participant’s: demographics, household information, mosquito avoidance behavior measures, general knowledge, level of concern, and perceptions of the seriousness of several mosquito-borne diseases, and responsibility for mosquito control measures.

All five diseases included in this pilot survey have been documented in humans in the United States, and in the case of CHIKV, DENV, WNV and ZIKV, domestically acquired specifically in Alabama (Table 1).

Surveys were returned to Auburn University’s School of Forestry and Wildlife Services and entered into a database for review and further analysis. Knowledge, concern and seriousness questions used 7-point Likert scales while avoidance behavior utilized frequency measures. Analysis is primarily descriptive. The output for this paper was generated using SAS 9.4, Copyright© [2002–2012] SAS Institute Inc. Visualizations were created using Microsoft Excel.

## Results

A total of 126 usable surveys were returned and are included in this analysis for a response rate of 15% when undeliverable surveys were deducted. Participants are largely representative of the underlying population demographics of their geographic area. Referring to the Census Quick Facts, July 1, 2017 population estimates [24] for Mobile Alabama. The survey participant population is slightly older than the census population. Given the similarity to the census demographic figures and budget constraints further non-response bias checks were not conducted. On average, the number of household occupants was 2.4 and 85% indicated they owned their home.

### Mosquito risk

**Fig. 1:**
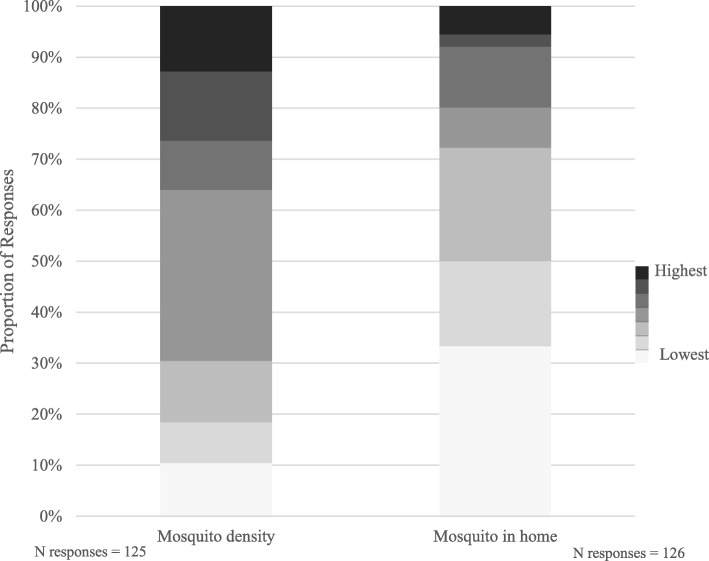
Participant’s reported mosquito density around home and frequency mosquito in their home, last 30 days

At the time of survey completion, 73% of participants reported being bitten by a mosquito in the last 30 days. Participants were asked to rate their perceptions of the level of mosquito density near their home during the last month on a scale from very low [1], to moderate [4], to very high [7]. Approximately 18% reported the two lowest categories combined very low to low density, 33% reported the middle moderate and 26% reported the two highest categories combined high to very high density (Fig. 1). Respondents were also asked to rate the frequency of seeing mosquitos in their home on a 7 point scale from almost never (33%), up to a few times a month (17% & 22%), up to a few times a week (8% & 12%), up to every day (2% & 6%) (Fig. 1). More than two-thirds (67%) of participants reported having screens on windows they open at their home and about three quarters (74%) reported having an open deck or unscreened porch at their home.

Assessing type and frequency of outdoor activities is informative in estimating someone’s risk of encountering a mosquito. We provide a breakdown of the frequency participants indicated doing various outdoor activities during the months, May–August (Table 3). On average, walking, relaxing and cooking outdoors were the most common outdoor summer activities. Relaxing (20%) and walking outdoors (31%) were the most frequently reported activities that participants reported doing outdoors almost everyday.

**Table 3:** Frequency in percentage of outdoor activities during summer months (May–August)

	Never	A few times a month or less	A few times a week	Almost everyday
Bird watching	89	9	1	1
Cooking outdoors	23	49	20	8
Fishing	58	35	7	0
Gardening	29	35	23	13
Hiking in woods	81	16	3	0
Horseback riding	97	3	1	0
Hunting	89	11	0	0
Relaxing	13	32	34	20
Running	72	19	5	4
Outdoor sports	64	28	6	3
Walking	8	29	31	31
Other outdoor activities	23	50	19	8

### Mosquito-borne disease knowledge, concern and perceptions of seriousness

Almost all participants, 94% reported hearing of ZIKV before receiving this survey. Participants were asked the level of their current knowledge regarding the five mosquito transmitted diseases. DENV and CHIKV were the two mosquito-borne diseases participants reported being the least familiar with each rating 88% less than knowledgeable (Table 4). MAL and WNV had 50% or more reporting that they were less than knowledgeable with only ZIKV reported at 52% knowledgeable or above. Participant knowledge of mosquito breeding preferences was high regarding some items (standing water (98%), bird baths (76%), old tires (71%)) but decreased significantly for some other common breeding areas (empty containers (51%) and rain gutters (61%).

**Table 4:** Level of knowledge, concern, and seriousness of each disease in percentages

Current knowledge of the following diseases		Not knowledgeable at all	Somewhat knowledgeable	Knowledgeable	Very knowledgeable	Extremely knowledgeable
	chikungunya virus	72	16	7	0	5
	dengue virus	66	22	5	2	4
	malaria	16	42	26	8	7
	West Nile virus	14	36	31	8	11
	Zika virus	10	39	33	7	12
Concern you or a family member will contract disease		Not Concerned	Minimally concerned	Concerned	More than concerned, but not extremely	Extremely Concerned
	chikungunya virus	35	30	20	0	15
	dengue virus	36	29	21	0	15
	malaria	34	32	19	2	13
	West Nile virus	16	38	24	3	18
	Zika virus	17	30	28	4	21
How serious do you feel an infection would be?		Not serious	Minimally serious	Serious	More than serious, but not extremely serious	Extremely serious
	chikungunya virus	3	13	39	10	38
	dengue virus	4	10	36	12	38
	malaria	4	8	34	14	42
	West Nile virus	3	11	30	17	40
	Zika virus	4	16	31	8	42

Concern expressed about potentially contracting one of the diseases had interesting dispersions. Almost half or more of all respondents indicated they were not concerned at all or minimally concerned about any of the diseases (47–66%). About a fifth to a quarter (19–28%) registered that they were concerned about they or a family member would contract any of the diseases. A small but significant percentage of participants (13–21%) reported being extremely concerned about contracting any of the diseases (Table 4). Participants indicated they were most concerned about contracting ZKV (21%) followed closely by WNV (18%). Nearly two-thirds of respondents (65–66%) were less than concerned about CHIKV, DENV and MAL.

Participants were asked how serious they thought an infection from each of the five diseases of interest would be should they or a family member become infected. Participants overwhelmingly reported (79–90%) that they thought all the diseases were serious to extremely serious if infected (Table 4). Nearly four in ten respondents felt that each of the diseases were extremely serious for their health.

### Mosquito avoidance

Two questions asking about participant’s activities to reduce mosquitoes around their home and prevent a mosquito bites showed participants are very knowledgeable about a variety of effective mosquito reduction and avoidance practices (Table 5). Dumping water from containers (54%) and flowerpots (58%) or treating bird baths (28%), checking boats or other large items (28%) and dumping water from kid’s toys (26%) were the most frequently reported activities occurring ‘a few times a week’ or more. The least frequently reported activities were checking rain gutters (71%) and using a bug lamp or zapper (77%) occasionally or never doing these activities.

**Table 5:** Mosquito avoidance and reduction practices in percentage

		Never/occasionally	A few times a month	A few times a week	Everyday
Activities to reduce mosquitoes around home	Replaced or treated water in a bird bath or fountain	56	16	18	10
	Used a bug lamp/zapper	77	7	8	7
	Checked boats and other large items for standing water	52	20	17	11
	Checked and cleaned gutters	71	17	9	4
	Cleared back overgrown shrubs/trees	30	49	18	3
	Dumped water from other containers that held water	17	29	42	12
	Dumped standing water from flower pots	14	28	41	17
	Dumped water from kid’s toys	58	19	13	10
	Mowed lawn	6	72	19	3
Activities to prevent a mosquito bite	Wear a long sleeve shirt	52	25	16	7
	Wear long pants	34	23	20	23
	Use a repellant without DEET	73	15	10	2
	Use a repellant with DEET	49	26	20	6
	Minimize time outside in the morning	43	19	18	20
	Minimize time outside in the evening	35	21	19	26
	Avoid areas where mosquitoes are present	34	17	18	32
	Burn Citronella candles	69	15	10	6
	Other	47	32	11	11

On average participants reported the most common activities they did to avoid a mosquito bite were ‘Avoiding areas where mosquitoes are present’ (50%) and ‘Minimize time outside in the evening’(48%) at least a few times a week. Highly effective mosquito bite avoidance activities like ‘wearing a long sleeve shirt (77%) or using a repellent with DEET (75%) were reported as being utilized less than a few times a month.

### Beliefs and impact

Participants are split about the impact their actions to control mosquitoes have on both their own home and their surrounding neighborhood. A little more than half of the responses (53%) indicated that people perceive their actions to have ‘some impact’ on the mosquito population around both their homes or neighborhoods. There is a small group or participants who perceive their actions to have a very little (14%) and another third (33%) who perceive a very significant impact around their homes and neighborhoods. Additionally, participants believe they (77%) along with their local government (69%) and health department (78%) are responsible for mosquito control efforts.

## Discussion

In this study, we conducted a survey to evaluate citizen knowledge, attitudes, and practices as they relate to mosquitoes and mosquito-borne diseases found in the southeastern United States.

### Mosquito risk

Our results indicate moderate densities of mosquitoes near homes in Mobile, as well as a high frequency of encounters with mosquitoes in residents’ homes (Fig. 1). The prevalence of mosquitoes in residential areas suggests that more emphasis on vector control is necessary to prevent risk of exposure to mosquito-borne pathogens. However, factors such as varying ecologies and increasing prevalence of insecticide resistance among mosquitoes make the task of vector control all the more daunting [9, 22].

Mosquitoes from the three main genera of public health concern, *Aedes*, *Anopheles* and *Culex*, have different ecologies and life-history strategies, complicating vector control strategies that target breeding sites or temporal avoidance of mosquitoes. Mosquitoes of the *Anopheles quadrumaculatus* complex, which are thought to be the only mosquitoes in the US capable of transmitting malaria, tend to breed in swampy habitats which are quite common in the Mobile Bay area [25]. *Culex* mosquitoes, which are the primary vectors of WNV, also often breed in man-made containers, though they will also breed in tree holes, rock pools, and sewage overflows [26]. Finally, the mosquitoes *Aedes aegypti*, *Aedes albopictus* and *Aedes japonicus*, which vector a suite of viruses and parasites including CHIKV, DENV, WNV and ZIKV, are diurnal feeders which prefer to breed in man-made containers, such as tires and flower pots [27–29]. Furthermore, *Aedes* mosquitoes tend to bite during the day, while *Culex* and *Anopheles* mosquitoes feed at dawn, dusk and nighttime [26]. This variety of ecologies necessitates a multitude of coordinated approaches to reduce mosquito vector populations. Improving mosquito burden around resident’s homes can be accomplished by encouraging residents to us unimpaired screens over windows and porches as well as clearing areas where mosquitoes prefer to live and breed.

The process of vector control is further complicated by the growing prevalence of insecticide resistance among *Aedes, Anopheles* and *Culex* mosquitoes [30–32]. Long-term and improper use of insecticides such as pyrethroids by commercial and governmental entities have led to the development of so called knockdown resistance (KDR) genes among mosquito populations around the world [30, 33]. While there are many efforts to create new insecticides to replace those to which the mosquitoes have adapted, future vector control strategies would be wise not to rely too heavily on insecticide use [22]. Instead, future mosquito control programs should integrate targeted insecticide use and reduction of mosquito habitat with public education initiatives [20].

### Mosquito-borne disease knowledge, concern and perceptions of seriousness

The results of our survey suggest that Mobile Bay area residents consider themselves more knowledgeable and more concerned about ZIKV and WNV than CHIKV or DENV. This perception may have been colored by the timing of the survey, which was released at the tail end of the 2014–2016 ZIKV epidemic in the Americas. ZIKV was often in the news at the time, the news was likely the source of residents’ knowledge/concern. This is supported by a comparatively high level of public knowledge/concern surrounding WNV, another virus that has appeared in the news much in recent history. While MAL and DENV are very rare still, CHIKV is relatively common in the Mobile area [14]. The fact that there are significant numbers in the extreme ends of the scale, ‘Not concerned’ at all and the ‘Extremely concerned’, indicates that more information is needed on the likelihood of exposure to these diseases. DENV and CHIKV, while commonly reported in US Gulf States such as Florida and Texas, are not as widely discussed in the media and thus the general public is not as educated about the threats they pose. Furthermore, knowledge regarding MAL might be due to its larger global discussion [34]. One potential reason for lack of concern may be because CHIKV and DENV like other flaviviruses (including ZIKV and WNV) do not always present as severe illness or may just feel flu-ish and not seek medical care [35]. In the majority of cases (up to 80% for ZIKV) infected individuals are asymptomatic [36]. The lack of physical symptoms may allow these viruses to go largely undetected.

The high level of knowledge about mosquito breeding preferences, standing water, old tires was also likely due to the local media coverage of the ZIKV epidemic at the time the survey was administered. The reported knowledge of mosquito breeding preferences emphasized many opportunities for improved education and outreach to make residents aware of the importance of a varied vector control approach because of the differences in preference by each mosquito species.

### Mosquito avoidance

The public health campaigns around the ZIKV epidemic were very effective, people did listen and understand the information disseminated by public health authorities and the media. Though the ZIKV epidemic has faded into the background, Gulf Coast and Mobile Bay region remain at threat from mosquito-borne pathogens. The mosquito avoidance activities reported in Table 5 are reflective of a population very aware of strategies to mitigate mosquito bites. This information also shows an opportunity to increase community awareness about the utility of a multi-pronged approach to mosquito avoidance. No single activity will eliminate mosquitoes or prevent bites because of the diversity of mosquito habitat, breeding and active periods.

### Beliefs and impacts

The data collected in this pilot survey shows that participants believe their actions can have an impact on the burden of mosquitoes in and around their homes. This presents an opportunity for public health official to engage with and equip the public with the knowledge and resources to reduce the risk of transmission of mosquito-borne pathogens.

### Strengths and limitations

This study provides data on human behaviors and perceptions of mosquitoes and mosquito-borne pathogens that can help refine and focus public health efforts, vector control efforts and public health educational materials. This survey was administered at the beginning of the summer and it is possible that residents were out of town when the survey and reminders were sent, limiting participation. This survey was also administered on the heels of the ZIKV outbreak and the intense media coverage at the time possibly skewed resident’s typical knowledge of mosquitoes and mosquito-borne disease. The response rate of the survey was limited which may reduce representativeness of the findings. Conducting mosquito trapping and surveillance activities at the residents of the participants would be needed to more strongly correlate disease risk with survey findings. Future work investigating different physiographic regions around the gulf where mosquito presence and disease risk likely to vary will provide more insight into the prevalence of mosquito species in the region.

## Conclusion

The results of this survey show strong evidence that residents of the Mobile Bay area in Alabama are aware of mosquitoes in and around their homes and that they are aware of at least some of the five mosquito-borne diseases included in this survey. Respondents overwhelmingly consider the health impacts from these diseases as serious. However, their self-reported knowledge of the different diseases is variable and their level of concern about they or their family contracting the diseases is bimodal, with some not concerned at all about them while others are extremely concerned. Clearly additional outreach on the different diseases, the likelihood of contraction, and the seriousness to different populations is needed. Participants are willing to take actions to reduce mosquito populations and are willing to behave in ways that reduce their exposure to mosquitos. The participants included in this analysis feel very empowered and responsible for mosquito control in and around their homes and neighborhoods and this presents a prime opportunity for education and outreach efforts. Capitalizing on the knowledge and the mosquito reduction and avoidance activities already being performed can provide opportunity for enhanced education and awareness. Improving education so local resident concern is appropriately placed for the vectors and diseases found in their area will allow residents to strategically engage in mosquito avoidance and reduction activities appropriate for the ecology of the vectors in their area. Outreach activities from the local government and health department who are considered very trustworthy by the respondents to improve education and awareness specific to these diseases and vectors would be an appropriate next step.

The results of this survey can assist in identifying and correcting misconceptions and misinformation about mosquito activity and the effectiveness of interventions and avoidance efforts, will be key for strengthening public health and community outreach activities.

## Additional file

Additional file 1: Mobile Mosquito Survey Booklet. This file is the questionnaire used for this study. (PDF 277 kb)

## Data Availability

The datasets used and/or analyzed during the current study are available from the corresponding author on reasonable request.

## Abbreviations

CHIKV: Chikungunya virus
DENV: Dengue fever virus
MAL: Malaria
WNV: West Nile virus
ZIKV: Zika virus
