# Supplementary material for: Perceptions and practices of mosquito-borne diseases in Alabama – is concern where it should be?
Source: BMC Public Health. 2019 Jul 23;19:987. doi: 10.1186/s12889-019-7308-x (PMC6652104; doi:10.1186/s12889-019-7308-x)
Supplement: Supplementary file 1 — Mobile Mosquito Survey Booklet. This file is the questionnaire used for this study. (PDF 277 kb) [file 12889_2019_7308_MOESM1_ESM.pdf]

2016 Mobile Bay Study on Mosquito-Transmitted Disease

Zika, West Nile, Chikungunya, Dengue, Malaria

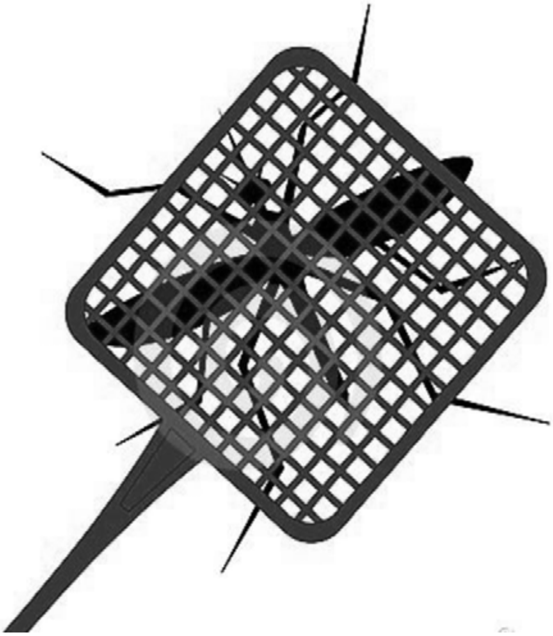

A Study By:
The School of Forestry and Wildlife Sciences
Auburn University

You and Your Household

1. **About how many years have you lived in your current residence?** ______ **years**

2. **Including yourself, how many people live in your residence?** ______ **people**

3. **What year were you born?** **1 9** ______

4. **What is your gender?** ○ **Female** ○ **Male**

5. **Do you rent or own your home (house, apartment, condo)?** ○ **Rent** ○ **Own**

6. **Do you use either an air conditioner or swamp cooler as the primary method of cooling your home?**

   ○ **Yes** ○ **No**

7. **Do you have an open deck or unscreened porch?**

   ○ **Yes** ○ **No**

8. **Do you have any torn or broken window screens that mosquitoes could use to enter your home?**

   ○ **Yes** ○ **No**

9. **Approximately when was your home built?** ______

10. **Do you have any pets/animals at your home?** ***(Check all that apply)***

- ☐ Cats
- ☐ Dogs
- ☐ Birds
- ☐ Chickens
- ☐ Other ______________________
- ☐ Other ______________________

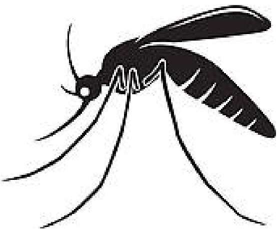

11. **Do you have a birdfeeder at your home?**

   ○ **Yes** ○ **No**

Mosquitoes and You

12. In the past 30 days, have you been bitten by a mosquito near your home?

○ **Yes** ○ **No**

13. Please rate the level of mosquito density near your home during the last 30 days.

1	2	3	4	5	6	7
Very low			Moderate			Very high
○	○	○	○	○	○	○

14. How frequently have you seen a mosquito inside your home during the last 30 days?

1	2	3	4	5	6	7
Almost never		A few times a month		A few times a week		Every day
○	○	○	○	○	○	○

15. Are you retired? ○ **Yes** ○ **No**

16. Are you currently employed? ○ **Yes** ○ **No (If no, skip to question #20)**

17. Do you work within approximately 1 mile of a working/industrial waterfront?

○ **Yes** ○ **No**

18. Do you work with livestock (cows, pigs, chickens, other)?

○ **Yes** ○ **No**

19. Approximately what percent of your time working is spent outdoors?

○ **0%** ○ **25%** ○ **50%** ○ **75%** ○ **100%**

20. Please rate the frequency with which you have encountered mosquitoes <u>while working</u> during the last <u>30 days</u>.

1	2	3	4	5	6	7
Almost never		A few times a month		A few times a week		Every day
○	○	○	○	○	○	○

21. Please indicate the frequency with which you perform the following outdoor activities during the summer (<u>May – August</u>).

	1	2	3	4	5	6	7
	Never		A few times a month		A few times a week		Every day
Walking	○	○	○	○	○	○	○
Running	○	○	○	○	○	○	○
Hiking in woods	○	○	○	○	○	○	○
Gardening	○	○	○	○	○	○	○
Relaxing outdoors	○	○	○	○	○	○	○
Cooking/eating outdoors	○	○	○	○	○	○	○
Horseback riding	○	○	○	○	○	○	○
Outdoor sports (football, soccer, etc.)	○	○	○	○	○	○	○
Fishing	○	○	○	○	○	○	○
Hunting	○	○	○	○	○	○	○
Bird watching away from your home	○	○	○	○	○	○	○
**Other outdoor activities:** ____________	○	○	○	○	○	○	○

22. Please indicate the frequency with which you have used the following practices to keep mosquitoes from biting you during the last 30 days.

	1	2	3	4	5	6	7
	Almost never		A few times a month		A few times a week		Every day
Wear a long sleeve shirt	○	○	○	○	○	○	○
Wear long pants	○	○	○	○	○	○	○
Use repellant without DEET	○	○	○	○	○	○	○
Use repellant with DEET	○	○	○	○	○	○	○
Minimize time outside in the morning	○	○	○	○	○	○	○
Minimize time outside in the evening	○	○	○	○	○	○	○
Avoid areas where mosquitoes are present	○	○	○	○	○	○	○
**Other:** ____________	○	○	○	○	○	○	○

23. On average, do your friends practice more or less of the types of precautions mentioned in question 22 than you do?

○ **More** ○ **About the same** ○ **Less**

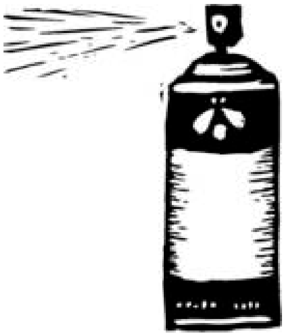

24. How would you rate your current knowledge of the following mosquito-transmitted diseases?

	1	2	3	4	5	6	7
	Not knowledgable at all			Knowledgable			Extremely knowledgable
Zika virus	○	○	○	○	○	○	○
West Nile virus	○	○	○	○	○	○	○
Chikungunya	○	○	○	○	○	○	○
Dengue fever	○	○	○	○	○	○	○
Malaria	○	○	○	○	○	○	○

25. How concerned are you that you or a family member will contract the following illnesses in the Mobile Bay area?

	1	2	3	4	5	6	7
	Not concerned at all			Concerned			Extremely concerned
Zika virus	○	○	○	○	○	○	○
West Nile virus	○	○	○	○	○	○	○
Chikungunya	○	○	○	○	○	○	○
Dengue fever	○	○	○	○	○	○	○
Malaria	○	○	○	○	○	○	○

26. If you were infected with one of the following, how serious do you feel it would be for your health?

	1	2	3	4	5	6	7
	Not serious at all			Serious			Extremely serious
Zika virus	○	○	○	○	○	○	○
West Nile virus	○	○	○	○	○	○	○
Chikungunya	○	○	○	○	○	○	○
Dengue fever	○	○	○	○	○	○	○
Malaria	○	○	○	○	○	○	○

Zika Virus

27. Before receiving this survey, had you ever heard of Zika virus?

○ **Yes** ○ **No**

28. Before receiving this survey, were you aware that Zika virus was transmitted to people by mosquitoes?

○ **Yes** ○ **No**

29. Which of the following animals do you believe can be infected with Zika virus?*(Check all that apply)*

☐ Birds ☐ Deer ☐ Don't know
☐ Cats ☐ Horses
☐ Dogs ☐ All mammals

30. Who are the people you believe are most vulnerable to Zika virus? *(Check all that apply)*

☐ Children ☐ Pregnant women ☐ Don't know
☐ People over 55 ☐ Women planning to have a baby soon
☐ Immune compromised people ☐ Men planning to have a baby soon

31. Where did you get your information about Zika virus? *(Check all that apply)*

☐ Television___ ☐ Word of mouth___ ☐ Heath care provider___
☐ Newspaper ___ ☐ Magazine___ ☐ Brochure/pamphlet___
☐ Radio___ ☐ Web site___ ☐ Other __________________ ___

32. On the line after the categories in question 31, please write a number 1 and a number 2 to identify the <u>two sources</u> you believe are the <u>most trustworthy</u>.

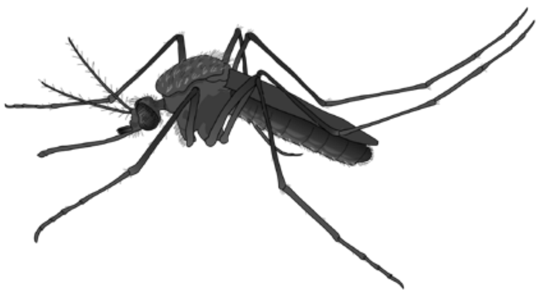

33. How frequently have you performed the following activities around your home to reduce mosquitoes during the last 30 days?

	1	2	3	4	5	6	7	
	Almost never		A few times a month		A few times a week		Every day	Does not apply to my home
Dumped standing water from flower pots	○	○	○	○	○	○	○	○
Dumped water from other containers that held water	○	○	○	○	○	○	○	○
Checked and cleaned gutters	○	○	○	○	○	○	○	○
Used a bug lamp/zapper	○	○	○	○	○	○	○	○
Cleared back overgrown shrubs/trees	○	○	○	○	○	○	○	○
Mowed lawn	○	○	○	○	○	○	○	○
Replaced or treated water in bird bath or fountain	○	○	○	○	○	○	○	○
Dumped water from kids' toys	○	○	○	○	○	○	○	○
Checked boats and other large items for standing water	○	○	○	○	○	○	○	○
Burned citronella candles	○	○	○	○	○	○	○	○
**Other:** ______________	○	○	○	○	○	○	○	○

34. On average, do your neighbors practice more or less of the types of precautions mentioned in question 33 than you do?

○ **More** ○ **About the same** ○ **Less**

35. Where do you believe mosquitoes breed? (*Check all that apply)*

☐ Flowing streams	☐ Flower pots	☐ Empty containers	☐ Rain gutters
☐ Streamside puddles	☐ Ornamental ponds	☐ Kids' toys	☐ Pools/Hot tubs
☐ Standing water	☐ Bird baths	☐ Old tires	☐ Boats

36. Have you removed or thrown away containers that held water in your yard in the last 30 days in order to reduce mosquitoes?

○ **Yes** ○ **No**

37. Have you had your yard professionally sprayed for mosquitoes in the last year?

○ **Yes** ○ **No**

38. Are you aware of any publicly funded spraying to reduce mosquitoes in your neighborhood in the last year?

○ **Yes** ○ **No**

39. How significant an impact do you think your personal actions will have in helping to reduce mosquitoes around your home?

**1**	**2**	**3**	**4**	**5**	**6**	**7**
No impact at all			Some impact			Very significant impact
○	○	○	○	○	○	○

40. How significant an impact do you think your personal actions will have in helping to reduce mosquitoes in your neighborhood?

**1**	**2**	**3**	**4**	**5**	**6**	**7**
No impact at all			Some impact			Very significant impact
○	○	○	○	○	○	○

41. Who do you feel is responsible for mosquito control? (*Check all that apply*)

- ☐ You___
- ☐ Local government___
- ☐ Local health department___
- ☐ State government___
- ☐ Federal government___
- ☐ Federal health organizations___
- ☐ Other ___________ ___

42. On the line after the category in question 41, please write a number 1 and a number 2 to identify <u>two groups</u> who you believe are the <u>most responsible</u>.

43. Looking up and down your block, what is the characteristic housing type on your street?

1	2	3	4	5	6	7
Mostly multi-unit housing			Mixed multi-unit & single family homes			Single family homes
○	○	○	○	○	○	○

44. Looking up and down your block, what is the general characteristic of <u>the landscaping</u> on your street?

1	2	3	4	5	6	7
Overgrown			Mixed care			Manicured
○	○	○	○	○	○	○

45. Looking up and down your block, do you see any abandoned homes and/or vacant lots?

1	2	3	4	5	6	7
Very few abandoned/ vacant			Some abandoned/ vacant			Lots of abandoned/ vacant
○	○	○	○	○	○	○

46. Looking up and down your block, do you see containers/trash that could hold water (for example: buckets, old tires, cans, etc.)?

1	2	3	4	5	6	7
Very few containers			Some containers			Lots of containers
○	○	○	○	○	○	○

47. Looking up and down your block, do you see landscaping containers that could hold water (for example: flower pots, bird baths, fountains, ponds, etc.)?

1	2	3	4	5	6	7
Very few containers			Some containers			Lots of containers
○	○	○	○	○	○	○

Demographic Information

48. What is your ethnicity?

- ○ American Indian
- ○ Black/African American
- ○ Latino
- ○ Asian
- ○ White/Caucasian
- ○ Other ____

49. What is your marital status?

- ○ Single
- ○ Married
- ○ Divorced
- ○ Widowed

50. What is your highest degree or level of school completed?

- ○ Did not complete high school
- ○ High School Diploma or GED
- ○ Some college, but no degree
- ○ Other ____
- ○ Associate degree
- ○ Bachelor degree
- ○ Graduate or professional degree

51. Please select the circle that corresponds to your household income for 2015. This information is only used to compare groups within this survey.

- ○ Less than $14,999
- ○ $15,000 – $19,999
- ○ $20,000 – $24,999
- ○ $25,000 – $34,999
- ○ $35,000 – $49,999
- ○ $50,000 – $74,999
- ○ $75,000 – $99,999
- ○ $100,000 – $149,999
- ○ $150,000 or more

Please fold this survey along the dotted line and return it to the School of Forestry and Wildlife Sciences at Auburn University in the self-addressed, stamped envelope provided.

THANK YOU FOR PARTICIPATING IN THIS STUDY!

Please provide any additional comments here.
